# COVID-19 Viral Pneumonia Complicated with Acute Pulmonary Embolism: A Descriptive Study

**DOI:** 10.1155/2021/6649086

**Published:** 2021-01-16

**Authors:** Hoda Salah Darwish, Mohamed Yasser Habash, Waleed Yasser Habash

**Affiliations:** ^1^Radiology Department, Faculty of Medicine, Suez Canal University, Ismailia, Egypt; ^2^Dallah Hospital, Riyadh, Saudi Arabia; ^3^Faculty of Medicine, Kasr Alainy Cairo University, Cairo, Egypt; ^4^Faculty of Medicine, New Giza University, Giza, Egypt

## Abstract

**Objective:**

To evaluate acute pulmonary embolism in patients with 2019 novel coronavirus (COVID-19) pneumonia using pulmonary CT angiography. *Subjects and Methods*. From 95 symptomatic patients confirmed with COVID-19 by RT-PCR from 1 May to 14 July 2020 in Dallah Hospital, Riyadh, Saudi Arabia, CT angiography was done for 25 patients suspected to have pulmonary embolism and have no contraindication for contrast study. 11 cases of them showed CT evidence of acute pulmonary embolism (PE). Retrospectively, CT angiography was analyzed and detailed findings were recorded. This study was approved by the Institutional Review Board of our hospital, and the patient consent was waived.

**Results:**

The mean age of the included patients was 49 ± 11 years; the youngest was 22 years, and the oldest was 64 years. Common symptoms in the 25 cases that underwent CT angiography were fever that was noted in 21/25 cases (84%), shortness of breath in 18/25 cases (72%), cough in 16/25 cases (64%), and severe myalgia/body fatigue in 13/25 cases (52%). The less common symptoms were sore throat in 11/25 cases (44%) and headache in 10/25 cases (40%). Regarding CT findings, 4/25 cases (16%) had unilateral lung disease and 21/25 cases (84%) had bilateral disease, with slight predilection for the right lower lobe (10/25 cases, 40%). Pure ground-glass opacity (GGO) was seen in 13/25 cases (52%), and GGO with consolidation was seen in 12/25 cases (48%). Common accompanying CT signs included crazy paving stone sign in 15/25 cases (60%) and air bronchogram in 12/25 cases (48%). From the 25 patients that showed respiratory deterioration and elevated serum D-dimer level, 11 cases confirmed to have acute pulmonary embolism, while 14 cases showed negative result for pulmonary embolism. 6/11 were male and 5/11 were female. Pulmonary embolism was diagnosed at a mean of 21 days from symptom onset. Unilateral acute pulmonary embolism was seen in 3/11 cases, while 8/11 cases showed bilateral distribution. Among 11 cases with acute pulmonary embolism, no emboli at the central level could be seen, but 3 cases showed pulmonary embolism at the lobar level, 3 cases at the segmental level, and 5 cases at lobar, segmental, and subsegmental levels.

**Conclusion:**

In patients with confirmed COVID-19, we should maintain a high suspicion for its thromboembolic complications such as acute pulmonary embolism that was mainly diagnosed at the end of 3rd week from symptom onset. We suggest that whenever a CT evaluation of the parenchymal involvement of COVID-19 pneumonia is performed, a simultaneous evaluation of the pulmonary arteries is also essential in order to identify early signs of associated pulmonary embolism.

## 1. Introduction

An outbreak of pneumonia of unknown etiology occurred in Wuhan, China, in December 2019, and 2019 novel coronavirus disease (COVID-19) was responsible [1]. The WHO declared it as a pandemic on March 11, 2020, in the following weeks; the disease has swept rapidly across most of the countries of the world, causing a global health emergency [2].

It is thought that viral spreading beyond the respiratory system to other organ systems in the 2nd week of the disease course (correlating with clinical worsening) leads to increased immune-mediated injury and hypercoagulability [3]. Some authors reported that acute pulmonary embolism is a cause of clinical deterioration in viral pneumonia [4, 5].

Han et al. reported disturbed coagulation function in patients infected with SARS-CoV-2 as compared to healthy controls, including elevated D-dimer, fibrin/fibrinogen degradation products, and fibrinogen levels [6]. Also, there is a positive correlation between elevated D-dimer levels on admission and in-hospital COVID-19 mortality [7, 8].

To date, most radiologic literature has focused on the distinctive chest CT abnormalities caused by COVID-19, including peripheral basal-predominant areas of ground-glass opacities and/or consolidation, often in a bilateral distribution [9].

However, emerging research has demonstrated that the inflammation and hypoxemia caused by COVID-19 may result in deranged coagulation parameters and increased risk of thromboembolic complications, which are associated with significant morbidity and mortality [10, 11]. They suggested that as patients with COVID-19 are admitted for treatment and isolation, it is important to follow prophylactic measures to prevent venous thromboembolism. In this condition, respiratory deterioration with other clinical evidence of venous thrombosis should raise suspicion for pulmonary embolism [12].

In our retrospective study, we focus on the development of acute pulmonary embolism as a related complication in patients with COVID-19 viral pneumonia.

## 2. Subjects and Methods

This study was approved by the Institutional Review Board of our hospital. The requirement for informed patient consent was waived by the ethics committee.

Out of 95 positive cases of COVID-19 in Dallah Hospital, Riyadh, Saudi Arabia, CT angiography was done for 25 patients suspected to have pulmonary embolism and have no contraindication for contrast study. 11 of them who showed CT evidence of acute pulmonary embolism were admitted to the ICU.

All CT scans were performed using a CT scanner (Light Speed™ 7.X CT scanner, GE Medical Systems, USA). Gantry position rotation period was 0.4 s, with an X-ray tube voltage of 140 kV and a current of 230–350 mA. Craniocaudal image acquisition of the entire thorax was carried out with a collimation of 1.25 mm (pitch of six) and a reconstruction increment of 0.8 mm. Mean scan time was 2.30 s. All patients underwent CTPA after administration of 100 ml standard contrast media (Xenetix 350 mg/ml, Guerbet, France) with an injection rate of 4.5–5 ml/s via a 16-gauge catheter inserted into the antecubital vein. Contrast bolus was followed by a 10 ml saline flush using Enhanced Xtream Injector (Nemoto Dual Shot Alpha (CiA425 Class IV/GE)), and delay was estimated using a semiautomatic bolus tracking system (SmartPrep, GE Medical Systems, USA) with a threshold of 80 HU for all examinations. ROI for bolus tracking was measured in the right atrium.

All reformations were performed by a senior CT technologist. From the axial CT data sets, multiplanar volume reconstruction (MPVR) and maximum intensity projection images were reformatted.

All images were sent to the workstation (Sectra, Sweden) and, retrospectively, CT angiography was analyzed and detailed findings were recorded.

We defined PE as a partial or complete intraluminal filling defect surrounded by the contrast medium and occupying the entire arterial vessel section [13]. The central, lobar, segmental, and subsegmental vessels of each lung were assessed for evidence of PE. We considered the main pulmonary artery, left and right pulmonary arteries, and interlobar arteries as the central vessel [14].

Presence or absence of emboli in the pulmonary artery or its branches was identified by using the nomenclature outlined by Remy-Jardin and Remy [13], which is based on the standard descriptions given by Boyden [15]; a 4-point scale was formulated to score different anatomical levels of pulmonary arteries of each lobe: (1) central (main, right and left, and interlobar), (2) lobar, (3) segmental, and (4) subsegmental levels.

### 2.1. Statistical Analysis

Statistical analyses were performed with SPSS for Windows software package (version 17.0, SPSS Inc.). Data were expressed as mean ± standard deviation (SD).

## 3. Results

Out of 95 cases confirmed to have COVID-19 viral pneumonia, 25 patients showed respiratory deterioration.

The mean age of the included patients was 49 ± 11 years; the youngest was 22 years, and the oldest was 64 years.

Common symptoms in the 25 cases that underwent CT angiography were fever that was noted in 21/25 cases (84%), shortness of breath in 18/25 cases (72%), cough in 16/25 cases (64%), and severe myalgia/body fatigue in 13/25 cases (52%). The less common symptoms were sore throat in 11/25 cases (44%) and headache in 10/25 cases (40%).

Regarding CT findings, 4/25 cases (16%) had unilateral lung disease and 21/25 cases (84%) had bilateral disease, with slight predilection for the right lower lobe in 10/25 cases (40%) and the middle lobe in 8/25 cases (32%). Pure ground-glass opacity (GGO) was seen in 13/25 cases (52%), and GGO with consolidation was seen in 12/25 cases (48%). Common accompanying CT signs included crazy paving stone sign in 15/25 cases (60%) and air bronchogram in 12/25 cases (48%).

In our study, acute pulmonary embolism was diagnosed at a mean of 21 days from symptom onset (range: from 19 to 24 days).

Unilateral acute pulmonary embolism was seen in 3/11 cases, while 8/11 cases showed bilateral distribution of acute pulmonary embolism.

**Figure 1:**
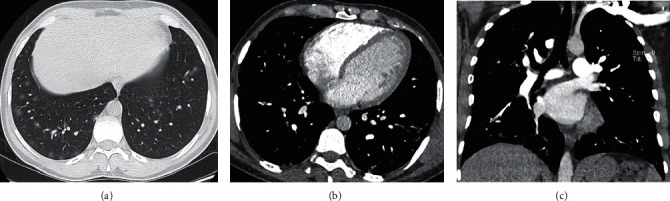
Images in a 38-year-old male with COVID-19 pneumonia complaining of fever and cough. (a) Axial unenhanced chest CT scan (lung window) obtained on day 5 after the onset of symptoms shows bilateral multiple patchy areas of ground-glass opacities more peripherally. (b) Axial CT pulmonary angiography scan. (c) Coronal reformatted images (mediastinum window) on day 19 after the onset of symptoms demonstrate filling defects (white arrow) within the segmental pulmonary artery branch supplying the lateral basal segment of the right lower lobe.

**Figure 2:**
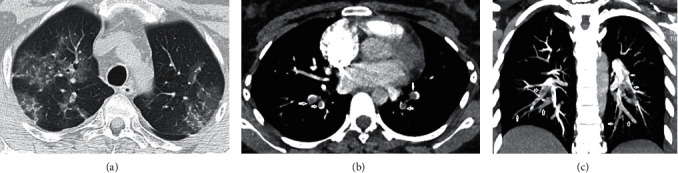
Images in a 22-year-old female with COVID-19 pneumonia complaining of fever, cough, and headache. (a) Axial unenhanced chest CT scan (lung window) obtained on day 6 after the onset of symptoms shows bilateral areas of central and peripheral ground-glass opacities and mixed density pattern. (b) Axial CT pulmonary angiography scan (mediastinum window). (c) Coronal thick maximum intensity projection slab of CT pulmonary angiography on day 24 after the onset of symptoms demonstrates filling defects (white arrows) in the left lower lobar as well as in both the right and left segmental/subsegmental pulmonary artery branches of the lower lobes.

**Figure 3:**
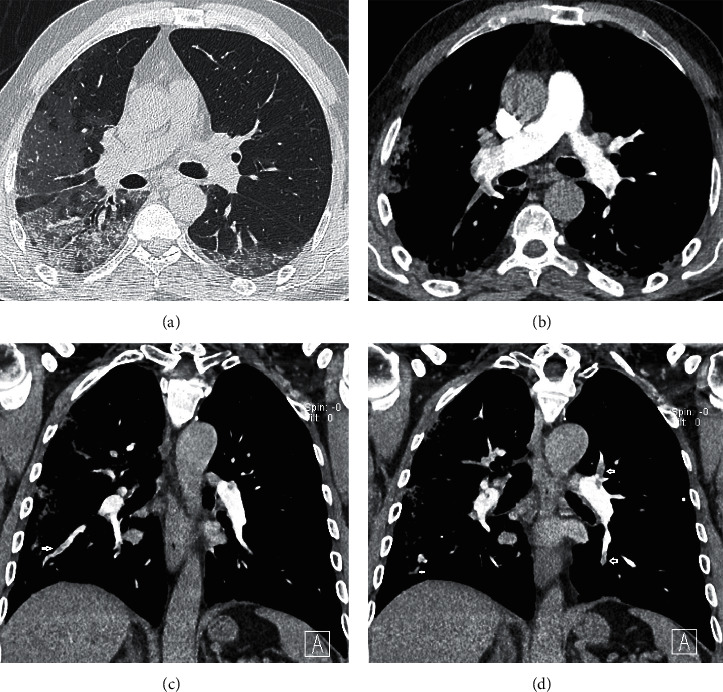
Images in a 64-year-old man with COVID-19 pneumonia complaining of body ache, fever, and cough. (a) Axial unenhanced chest CT scan (lung window) obtained on day 9 after the onset of symptoms shows bilateral areas of peripheral ground-glass opacities more at the right side with interlobular septal thickening (crazy paving sign), vascular sign, and air bronchogram sign being seen. (b) Axial CT pulmonary angiography scan. (c, d) Coronal reformatted images (mediastinum window) on day 24 after the onset of symptoms demonstrate multiple bilateral filling defects (white arrows) involving lobar, segmental, and subsegmental branches of the pulmonary artery.

Among the included 11 cases with acute pulmonary embolism, no emboli could be detected at the central level (main, right, left, and interlobar pulmonary arteries), but 3 cases showed acute pulmonary embolism at the lobar level, 3 cases at the segmental level (Figure 1), and 5 cases at lobar, segmental, and subsegmental levels (Figures 2 and 3).

The respiratory rate in the 11 cases with acute pulmonary embolism was 16/min–18/min, and oxygen saturation was 95%–93% on room air. Blood sampling of patients showed leucopenia, lymphopenia, and thrombopenia. Laboratory tests showed prolongation of the prothrombin time (range: 14.2–16.3) with elevated D-dimer level (1.56 mg/ml–8.8 mg/ml).

The 11 COVID-19 cases positive for pulmonary embolism were admitted to the ICU and followed by the cardiologist and intensivists. Echocardiography was not done for any of our cases.

## 4. Discussion

Several recent studies have explored the link between COVID-19 and hypercoagulability, suggesting that the most common laboratory test result abnormalities include elevated D-dimer levels and mild thrombocytopenia [16]. In our study, all our positive cases for acute pulmonary embolism showed high D-dimer level.

The most typical finding in patients with COVID-19 and coagulopathy is an increased D-dimer concentration, a relatively modest decrease in the platelet count, and a prolongation of the prothrombin time; the combination of thrombocytopenia, prolonged prothrombin time, and increased D-dimer level is suggestive of DIC [17], which was evident in our cases.

In our study, acute pulmonary embolism was seen in 11/95 cases (11.6%), which is less than other studies that showed more frequent pulmonary embolism in patients with COVID-19 and reported almost a quarter of the COVID-19 pneumonia patients [18]; this may be because of a small number of our study cases and also because of early diagnosis of positive cases and good treatment plans in our hospital. This may also be because CT angiography was done only in deteriorating patients (25/95 cases), with high D-dimer levels and high possibility for acute pulmonary embolism, and not in all COVID-19 pneumonia patients.

Some authors reported that lung injury and impaired gas exchange lead to increased production of proinflammatory cytokines, and contributing viral infection may result in vascular endothelium direct damage [19].

Another study demonstrated severe endothelial injury associated with intracellular virus and disrupted cell membranes, as well as extensive thrombosis, microangiopathy, alveolar capillary microthrombi, and more new vessel growth, compared with those lungs obtained at autopsy from patients who died from influenza [20].

Our study suggests that COVID-19 patients with severe clinical features such as respiratory deterioration and elevated serum D-dimer level may have associated acute pulmonary embolism and need contrast-enhanced CT angiography study for diagnosis.

Our results revealed that pulmonary embolism was noted at lobar, segmental, and subsegmental pulmonary artery levels with a clear central pulmonary artery (main, right, and left branches).

Imaging features of acute pulmonary embolism in our study in patients with COVID-19 are nonspecific and similar in appearance to those seen in other pathologic conditions, which were also reported by other authors [21].

### 4.1. Limitations of the Study

Our finding is preliminary in nature in our initial experience with COVID-19 cases. CT angiography was not systematically used for all COVID-19 patients, but only for deteriorating patients with high D-dimer levels, so we cannot assess the incidence of PE in COVID-19 patients. No lower limb venous Doppler ultrasound study was done for our patients to evaluate the presence of deep venous thromboembolism. In addition to a small number of our subjects, further study with more numbers is needed.

## 5. Conclusion

In patients with confirmed COVID-19, we should maintain a high suspicion for its thromboembolic complications such as acute pulmonary embolism that was mainly diagnosed at the end of 3rd week from symptom onset.

Whenever a CT evaluation of the parenchymal involvement of COVID-19 pneumonia is performed, a simultaneous evaluation of the pulmonary arteries is also essential in order to identify early signs of associated pulmonary embolism.

In any case, early anticoagulant therapy initiated in the early stages of infection will make the CT pictures much less striking compared to the first case studied in the literature and, therefore, the CT could maintain a role in the follow-up of pumonary embolsim in case of disappearnce of symptoms.

## Data Availability

The data used to support the findings of this study are available from the corresponding author upon request (darwish.hoda@yahoo.com).

## Abbreviations

COVID-19: 2019 novel coronavirus
NCP: Novel coronavirus pneumonia
CTA: Computed tomography angiography
GGO: Ground-glass opacity
WHO: World Health Organization
PE: Pulmonary embolism
RT-PCR: Real-time reverse transcription polymerase chain reaction
SOB: Shortness of breath
ICU: Intensive care unit
DIC: Disseminated intravascular coagulopathy.
